# Toxicity of *Piper hispidinervum* Essential Oil to *Callosobruchus maculatus* and Cowpea Bean Quality

**DOI:** 10.3390/plants13223148

**Published:** 2024-11-09

**Authors:** Maria Suely Siqueira Ferraz, Lêda Rita D’Antonino Faroni, Adalberto Hipólito de Sousa, Fernanda Fernandes Heleno, Marcus Vinicius de Assis Silva, Ernandes Rodrigues de Alencar

**Affiliations:** 1 Department of Agricultural Engineering, Federal University of Viçosa, Viçosa 36570-900, MG, Brazil; suely.ferraz17@hotmail.com (M.S.S.F.); marcus.assis@ufv.br (M.V.d.A.S.); ernandes.alencar@ufv.br (E.R.d.A.); 2 Center for Biology and Nature Sciences, Universidade Federal do Acre, Rio Branco 69920-900, AC, Brazil; adalbertohipolito@hotmail.com; 3 Serviço Autônomo de Água e Esgoto de Senador Firmino, Senador Firmino 36540-000, MG, Brazil; fernandafhg@gmail.com

**Keywords:** Piperaceae, bioinsecticide, *Vigna unguiculata*, bruchid, grain quality

## Abstract

Essential oils and their major compounds have been studied to protect stored grains, especially for the control of insects. In this context, this research aimed to investigate the fumigation and contact toxicities of the essential oil of *Piper hispidinervum* C. DC. (*Piperaceae*) (sin. *Piper hispidum* Sw.) to *Callosobruchus maculatus* adult individuals and the effect on insect progeny. We also assessed the essential oil’s effect on stored-cowpea quality. The fumigation bioassay used essential oil at 14.3, 57.1, 100.0, 142.9, and 185.7 µL/L of air, whereas the contact bioassay tested concentrations of 60, 80, 100, 120, and 140 µL/kg. Insect mortality was appraised after four days (fumigation) or one day (contact). In turn, oviposition and emergence rates were evaluated after seven (fumigation) or fifty (contact) days of storage. Grain quality was also analyzed after 50 days of storage. Safrole was confirmed as the primary compound of the essential oil. *P. hispidinervum* essential oil proved its fumigant and contact toxicities to *C. maculatus* adult individuals. The concentrations lethal to 50 and 95% of the population were, respectively, 91.23 and 242.59 µL/L of air (fumigation) and 101.51 and 208.52 µL/kg of cowpeas (contact). In both application forms, *C. maculatus* oviposition and progeny rates declined with the increase in the essential oil concentration. Furthermore, cowpea bean quality was preserved even at sublethal doses.

## 1. Introduction

Essential oils are derived from the secondary metabolism of aromatic plants and constitute a complex of volatile compounds with a strong odor [1]. EOs and their components are promising alternatives for protecting stored products [2,3,4,5,6,7,8], as they present insecticidal activities such as toxicity by fumigation [1,9,10], contact [11], and repellency [12,13,14]. They also negatively affect population growth and development rates [15] and reproduction [16] and can even change the behavior [9] and physiology of pests [9,15]. Essential oils are generally considered safe for the environment and humans [17,18,19].

The genus *Piper* (Piperaceae) is distributed in tropical and subtropical regions [20]. Some species have commercial value due to their potential for producing essential oils and the bioactivity of secondary compounds, which are employed by the food and pharmaceutical industries [21,22]. *Piper hispidinervum* C. DC. (*Piperaceae*) (sin. *Piper hispidum* Sw.) is a shrub endemic to the Amazon region, and it stands out among other aromatic species of the genus for its application in cosmetics and insecticides [23]. Studies have pointed to the effects of *P. hispidinervum* essential oil (PHEO) on insect pests of stored products, including toxicity (by fumigation and contact), repellency, and behavioral alterations [9,12,13]. 

The main compounds in PHEO are safrole, terpinolene, and E-β-ocimene [24]. Safrole (phenylpropanoid) is the most abundant [9,13,25], and it is industrially used to synthesize the insecticide synergist piperonyl butoxide (PBO) [26] and the fragrance fixative heliotropin [27].

Insect pests during storage lead to substantial qualitative and quantitative losses in grains. The weevil *Callosobruchus maculatus* Fabr. 1775 (Coleoptera: Chrysomelidae: Bruchinae) is the primary storage pest of *Vigna unguiculata* L. Walp. (Fabaceae) cowpeas [2]. Its life cycle has four development stages: egg, larva, pupa, and adult. During the larval phase, the weevil feeds on the grain nutritive reserves, thus potentially leading to a total loss within a few months due to its high reproductive capacity and short life cycle [28,29]. The insect attack depletes the nutritional content of the beans (protein, carbohydrates, tannins, phenols, and minerals), reduces their commercial value, and ultimately poses a food security threat, as many farmers subsist on this crop [29] 

Phosphine (PH_3_) is the fumigant insecticide most commonly used against insect pests in stored products [30,31]—in some countries, it is the only one allowed. Protective synthetic insecticides (pyrethroids and organophosphates) are also employed for managing insect pests. However, the excessive use of these substances has triggered the emergence of populations of different insect-pest species resistant to phosphine [32,33,34,35] and protective insecticides [36,37]. Applying insecticides with different active principles, like essential oils, can mitigate or delay the development of resistant populations, reducing the need for synthetic products [37,38]. Other techniques have been used, such as modified atmospheres. With the modification of the atmosphere, the physiological environment of the insects is altered, and, consequently, the infestation is controlled [39]. Among the new techniques for the control of insects in grains and by-products, cold plasma [40], gamma irradiation [41], and radiofrequency [42] stand out.

In light of the above, this study aimed to determine the toxicity (by fumigation and contact) of PHEO to *C. maculatus* in cowpea beans. It also sought to investigate the effects of PHEO on the oviposition and progeny rates of the insects and the quality of stored grains.

## 2. Materials and Methods

### 2.1. Insect Stock Colony

The insects were reared in cowpea beans under constant temperature (25 ± 2 °C), relative humidity (70 ± 5%), and a 24 h scotophase. They were confined inside 1.5 L glass flasks closed with a perforated plastic lid and internally lined with organza fabric to allow gas exchange. Adults of *C. maculatus* were kept for seven days so they could lay eggs. After that, the insects were removed from the container, and the cowpeas were stored until the emergence of generation F1. Every two days, the cowpeas were sieved to remove any emerged adults so as to guarantee the 48 h age control. This procedure was executed throughout the bioassays.

### 2.2. Characterization of the Cowpea Beans

The experiment used cowpea beans (variety BRS Guariba) cultivated in the municipality of Codó, Brazilian state of Maranhão (4°27′18″ S latitude, 43°53′09″ W longitude, and 43 m altitude). The beans had the following qualitative characteristics: pest infestation = 4%, water content = 12.3% w.b., germination = 98%, and bulk density = 787 kg/m^3^. After harvesting, the grains were stored at −18 °C to prevent microorganism contamination until further use.

### 2.3. Essential Oil Extraction

*P. hispidinervum* plant material was collected by the road BR 317, at the milepost 30 Km, Ramal Iquiri, in Rio Branco, state of Acre, in the Brazilian Amazon Rainforest (9°58′29″ S latitude, 67°48′36″ W longitude, and 153 m altitude). The collection took place in June 2017, in the mornings. The leaves were removed from the branches and partially dried out under ambient conditions. Then, they were taken to an oven at 36 °C for dehydration until a constant weight was reached. PHEO was extracted with a heating mantle, model 0321A28 (Quimis, Diadema, SP, Brazil), using a 5 L volumetric flask and a Clevenger-type apparatus. Next, the PHEO was parted from the emulsion by decanting in a separating funnel, using anhydrous sodium sulfate (99.0%, Synth, Brazil). Last, the PHEO was stored in an amber flask at 4 ± 1 °C.

### 2.4. Essential Oil Composition

PHEO was analyzed via gas chromatography coupled with mass spectrometry (GC-MS) (model QP2020 (Shimadzu, Tokyo, Japan)). The chromatographic conditions were as follows: SH-Rtx-5MS capillary column model (Shimadzu, Tokyo, Japan) with dimensions of 30 m length, 0.25 mm internal diameter, and 0.25 µm thickness; helium (99.999%, Air Products, Brazil) as the carrier gas at a flow rate of 1.17 mL/min; and an injector at 220 °C. The column temperature started at 60 °C and was increased by 2 °C min^−1^ up to 200 °C, and then by 5 °C min^−1^ to 250 °C, where it was kept for 1 min. The chromatograph was operated in a full-scan mode in a 1:20 split ratio. The total analysis time was 81 min. The resulting mass spectra were compared with those from the NIST-14 library and by calculating the Kovats index for a series of saturated alkanes (C7–C30) (49451-U, 99.0%, Supelco, Bellefonte, PA, USA).

The main constituents were identified by their retention index (RI) in relation to a homologous series of n-alkanes. Then they were confirmed by comparing the mass spectrum of the compounds with the NIST-14 spectral library.

### 2.5. Absolute Quantification of Safrole

The absolute quantification of safrole in PHEO was performed with a gas chromatograph with a flame-ionization detector (GC-FID) (model GC2014 (Shimadzu, Tokyo, Japan)). Five safrole solutions in methanol were injected into the device at 0.25, 0.50, 1.00, 1.50, and 2.00 mg/mL, in three repetitions. PHEO in methanol was also injected at 1 mg mL^−1^ (99.9%, Vetec, Duque de Caxias, Brazil), in triplicates for each repetition. The chromatographic conditions for quantifying safrole were the following: a DB-5 capillary column model (Shimadzu, Tokyo, Japan) with dimensions of 30 m length, 0.25 mm internal diameter, and 0.10 µm film thickness; nitrogen (99.999%, Air Products, São Paulo, SP, Brazil) as the carrier gas at a flow rate of 1.82 mL/min; an injector at 220 °C; a flame-ionization detector at 300 °C; and a 1:5 split ratio. The column temperature was initially set at 60 °C, then raised by 5 °C min^−1^ to 120 °C and held for 1 min. The total analysis time was 12 min.

### 2.6. Toxicity Bioassays and Rates of Oviposition and Progeny

This research determined fumigant and contact toxicities of PHEO to adults of *C. maculatus*. The bioassays were carried out under constant temperature (25 ± 2 °C), relative humidity (70 ± 5%), and scotophase (24 h). Initially, preliminary tests were conducted to estimate the concentrations leading to the highest and lowest mortality rates within a 5–95% range.

The fumigation tests were performed in six replications, employing PHEO at 14.3, 57.1, 100.0, 142.9, and 185.7 µL/L of air and a control treatment (only cowpea beans). The experimental units consisted of 300 mL glass flasks (6.7 cm diameter × 12.9 cm height) containing 200 g of cowpea beans and 50 non-sexed *C. maculatus* adult insects aged up to 48 h after emergence. The PHEO was applied on a filter paper (2.5 cm × 8.0 cm) wrapped in a metallic screen (12.3 cm × 5.6 cm × 0.5 cm, 4 mm mesh). A piece of organza-type fabric (15 cm × 15 cm) was placed vertically in the grains to prevent the direct contact of the insects with the essential oil. The flasks were closed with a metallic screw cap and sealed with parafilm (PM996, American, Miami, FL, USA). After four days of exposure, the insects were removed from the grains, and the number of dead and living individuals was counted.

The contact-toxicity bioassays used PHEO at 60, 80, 100, 120, and 140 µL/kg of cowpea beans and a control (pure acetone), in six replications. PHEO was diluted in acetone in order to homogenize the product distribution throughout the grain mass. The mixture volume applied was 400 µL per 200 g of cowpea beans, corresponding to 2 L/t of grains. The PHEO was sprayed with a double-action airbrush model BC 60 (Steula, Sao Paulo, SP, Brazil), which operated with an internal mixing system and a gravity deposit, at a working pressure of 1034.21 hPa. After the application, the insects were released into the grain mass. The experimental units consisted of 0.8 L glass flasks (8 cm diameter × 15 cm height) with 200 g of cowpea beans and 50 non-sexed *C. maculatus* adult insects, aged up to 48 h after emergence. The flasks were closed with organza-type fabric (15 cm × 15 cm) to keep the insects from escaping while allowing gas exchange. After the 24 h exposure period, the deceased and alive individuals were counted.

The cowpea beans used to determine *C. maculatus* oviposition and emergence rates post fumigation and contact-toxicity testing had been previously stored for 50 days, under constant temperature (25 ± 2 °C) and relative humidity (70 ± 5%). The oviposition rate was evaluated seven days after the mortality tests by quantifying the eggs in a 50 g cowpea bean sample. The emergence rate was determined by counting the total number of adult insects after 50 days of storage.

After the fumigation and contact-toxicity bioassays, the cowpea beans were stored for 50 days, when they had their quality appraised as a function of the PHEO concentration. The parameters evaluated were the following: moisture content (% w.b.), mass loss (%), germination (%), and bulk density (kg/m^3^) [43]. The experimental units were kept under controlled temperature (25 ± 2 °C) and relative humidity (70 ± 5%).

Moisture content was determined through the oven method, using 30 g of beans in triplicate for each of the six repetitions. These samples were put inside an oven with forced air convection and heating set at 105 ± 3 °C for 24 h. After that, the samples were weighed to determine the moisture content, and the results were expressed in wet basis (% w.b.). The mass loss during storage was established by weighing the grains in an analytical balance (model BK8000 (Gehaka, Sao Paulo, SP, Brazil)) before storage and afterward. The germination test employed eight samples of 50 grains for each repetition. Germitest paper was used as the substrate, which had been moistened with distilled water at 2.5 times the paper weight. The material was stowed in a germination chamber (Biomatic, Porto Alegre, RS, Brazil) at 25 ± 1 °C. The germinated grains were counted eight days later, and the resulting data were expressed as average germination percentage. Bulk density was gauged with a hectoliter weight scale with 250 mL capacity model 40 (Dellemolle, Caxias do Sul, RS, Brazil). The analysis was carried out in triplicate for each repetition, and the results were expressed in kg/m^3^.

### 2.7. Statistical Analysis

In the fumigation and contact-toxicity bioassays, insect mortality was corrected by Abbott’s formula [44]. Concentration–mortality data were subjected to Probit analysis (PROC PROBIT, SAS Institute, Cary, NC, USA, 2011). The oviposition and emergence rates of the insects and the qualitative variables of the stored cowpea beans were subjected to regression analysis as a function of the PHEO concentration. Graphs were plotted using Sigmaplot software version 12.5 (SPSS, Inc., Chicago, IL, USA).

## 3. Results

### 3.1. Essential Oil Composition

GC-MS was employed to establish the chemical composition and relative quantification of the compounds in the essential oil from *P. hispidinervum* leaves. The chromatographic analysis revealed six substances in the PHEO (Table 1), with safrole being the major one (93.0%), followed by bicyclogermacrene, n-pentadecane, spathulenol, p-cymen-8-ol, and (E)-caryophyllene (2.05, 1.60, 1.46, 1.20, and 0.69%, respectively). Safrole’s absolute concentration in the PHEO was accessed with the GC-FID, according to the compound retention time. The results showed that a PHEO solution in methanol at 1.00 mg/L^1^ contained 0.85 mg/L^1^ of safrole, representing 85% of the essential oil.

### 3.2. Toxicity Bioassays and Rates of Oviposition and Progeny

**Figure 1:**
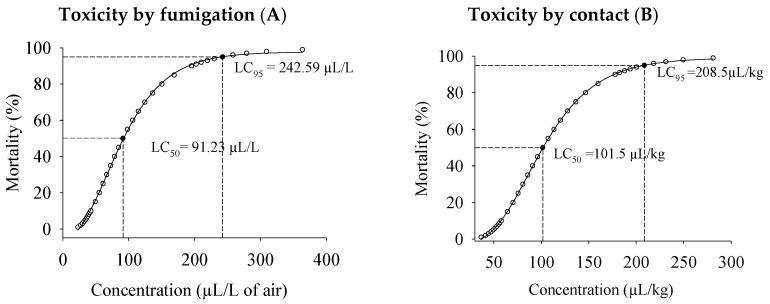
Toxicity by fumigation (**A**) and contact (**B**) of *P. hispidinervum* essential oil to adults of *C. maculatus* in cowpea beans.

The Probit model fitted the data based on PHEO concentration–mortality, as attested to by the low ꭓ^2^ value and the high *p*-value, in both the fumigant (ꭓ^2^ = 3.92, *p* = 0.27; Figure 1A) and contact (χ^2^ = 3.54, *p* = 0.31; Figure 1B)-toxicity bioassays. In the fumigation tests, the lethal concentrations for attaining 50 and 95% insect mortality (LC_50_ and LC_95_) were 91.23 (83.23–99.07) and 242.59 (208.90–298.24) µL/L of air, respectively (Figure 1A). The curve slope was 3.87 ± 0.36, indicating genetic heterogeneity within the *C. maculatus* population. As for the contact toxicity, the LC_50_ and LC_95_ were 101.51 (96.33–107.21) and 208.52 (183.33–250.76) µL/kg of cowpea beans, respectively (Figure 1B). The curve slope was 5.26 ± 0.51, implying genetic homogeneity within the insect population.

**Figure 2:**
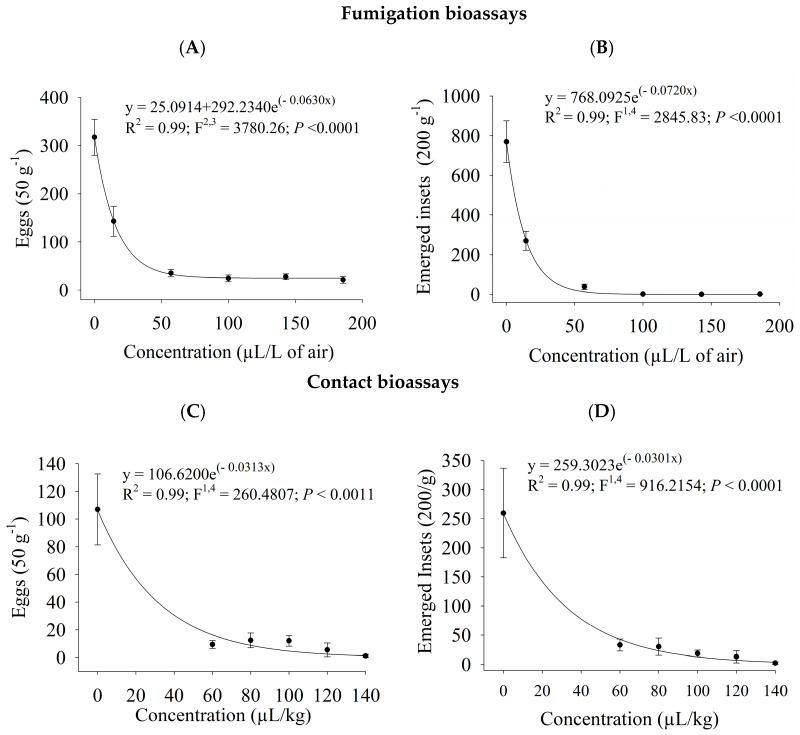
*C. maculatus* oviposition and progeny rates in cowpea beans treated with *P. hispidinervum* essential oil by fumigation (**A**,**B**) and contact (**C**,**D**).

The oviposition and emergence rates of *C. maculatus* decreased significantly as the PHEO concentration augmented (*p* ≤ 0.001) in the fumigation and contact bioassays. The exponential model had the best fits to the data on oviposition and emergence rates in both application forms (Figure 2). Fumigating cowpea beans with PHEO at 14.3 to 185.7 µL/L of air substantially lowered oviposition (54.9 ± 9.8 to 93 ± 2.2%; Figure 2A) and progeny rates (64.9 ± 6.2 to 100 ± 0.00%; Figure 2B). Also, the contact bioassays with PHEO at 60 to 140 µL/kg caused considerable reductions in oviposition (88.5 ± 5.0 to 98.9 ± 1.1%; Figure 2C) and progeny rates (87.3 ± 3.9 to 99.1 ± 0.8%; Figure 2D).

### 3.3. Quality Analyses of Cowpea Beans

**Figure 3:**
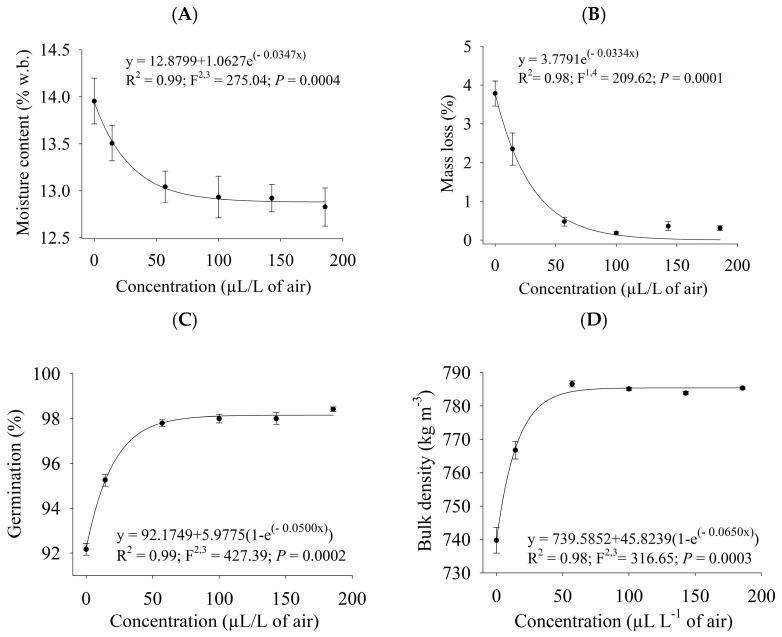
Quality analyses of grains fumigated at different *P. hispidinervum* essential oil concentrations. (**A**) moisture content (w.b.), (**B**) mass loss, (**C**) germination, and (**D**) bulk density.

**Figure 4:**
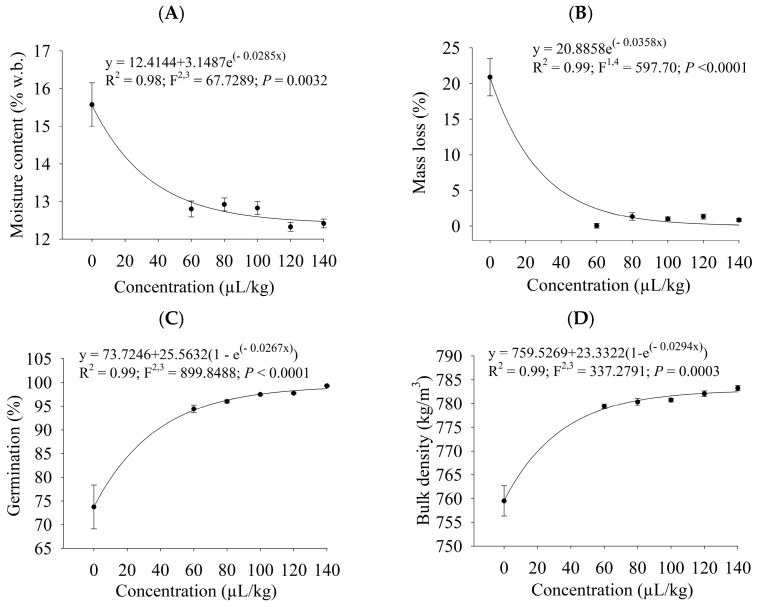
Quality analyses of grains sprayed at different *P. hispidinervum* essential oil concentrations. (**A**) moisture content (w.b.), (**B**) mass loss, (**C**) germination, and (**D**) bulk density.

After being stored for 50 days, cowpea beans fumigated or sprayed with different concentrations of PHEO were subjected to quality analyses (moisture content, mass loss, germination, and bulk density). All quality features varied with the increase in the PHEO levels (*p* ≤ 0.0040). The exponential model had the best fits to the data on all considered variables (Figure 3 and Figure 4). Grain moisture content (Figure 3A and Figure 4A), mass loss (Figure 3B and Figure 4B), germination (Figure 3C and Figure 4C), and bulk density (Figure 3D and Figure 4D) varied as a function of the concentration of PHEO in the fumigation and contact bioassays. Grain quality was preserved when higher PHEO concentrations were used. In fumigated grains, quality did not change at levels higher than 57.1 µL/L of air, whereas in sprayed beans, it remained unaltered above 60 µL/kg.

## 4. Discussion

PHEO proved effective in controlling *C. maculatus* adults and inhibiting its progeny development in cowpea grains treated by fumigation or contact. In addition, sublethal PHEO doses preserved cowpea quality in both application methods.

In general, although the PHEO fumigant toxicity bioassays showed an LC_50_ and LC_95_ of 91.23 and 242.59 µL/L of air, respectively, adult progeny reduced substantially, and grain quality was preserved at concentrations above 57.1 µL/L of air after four days of fumigation. In an investigation conducted by Oliveira et al. [13] to determine the toxicity of essential oils from six botanical species to *C. maculatus*, the LC_50_ of PHEO was only 41.46 µL/L of air two days after fumigation. However, in that case, no grains were used in the experimental units. On the other hand, the present research used 200 g of cowpea beans, considering that the grains can adsorb PHEO and act as a physical barrier to the fumigation process, thus reducing the essential oil toxicity. Paes et al. [46] studied the transport of allyl isothiocyanate in corn and observed the occurrence of sorption of this compound by the grains. According to Lu et al. [47], allicin, the major compound of the essential oil of *Allium sativum* L. (Amaryllidaceae), was adsorbed by wheat grains *Triticum* sp. (Poaceae) during fumigation and declined in concentration over time. The same was noticed for safrole, whose concentration in fumigated and stored cowpea grains was less than 1% of the initial value after five days [19]. In the application of PHEO for insect control, adsorption by the grains reduces the concentration of the major compound in the intergranular space. Therefore, it is necessary to increase the concentration of PHEO in order to obtain mortality percentages similar to those verified in the absence of grains.

Contact toxicity was also verified in the experiments. This result corroborates the works by Coitinho et al. [12] on PHEOs rich in safrole (94.7%), in which the authors observed toxicity by contact and ingestion to adult individuals of *Sitophilus zeamais* Mots. (Coleoptera: Curculionidae). They obtained an LC_50_ of 25 µL/kg of corn grains, considering an exposure of 48 h. Mossi et al. [48] detected a high topical toxicity of the essential oil of sassafras, *Ocotea odorifera* (Vellozo) Rohwer (Lauraceae), rich in camphor (43%) and safrole (42%), to *S. zeamais* adults, with an LD_50_ of 0.09 µL/cm^2^. In another study evaluating the insecticidal activity of safrole and isosafrole, the contact toxicity of these compounds was similar in adults of *S. zeamais* and *Tribolium castaneum* (Herbst) (Coleoptera: Tenebrionidae). Furthermore, those authors noticed that 16-day-old larvae had greater tolerance than the younger ones (12 and 14 days old) [49]. Thus, according to the studies abovementioned, it can be inferred that PHEO toxicity is linked to its constituents, especially safrole.

Safrole is the most abundant compound in PHEO, as proved in this and other investigations [9,13,24,25,50]. It is also the main component of the essential oils of *Piper mikanianum* (Kunth) Steudel [51], *Peperomia inaequalifolia* Ruiz & Pav (Piperaceae) [52], *Sassafras albidum* (Nutt.) (Lauraceae) [53], *Ocotea odorifera* (Vellozo) Rohwer (Lauraceae) [48], *Cinnamomum kanehirai* Hayata and *Cinnamomum micranthum* Hayata (Lauraceae) [54], and *Cinnamomum longepaniculatum* (Gamble) N. Chao [55]. Other PHEO compounds identified by other authors include pentadecane [50], bicyclogermacrene and (E)-caryophyllene [9,13,25,50], spathulenol [9,13,25], and p-cymen-8-ol [9,13].

Safrole is a substance easily found in spices and condiments, such as cinnamon, nutmeg, black pepper, and basil. It has also been widely used as a natural or synthetic flavoring agent. Most terpenoids and phenols found in essential oils have low toxicity to vertebrates, so they have been approved as flavoring agents in foods and beverages by the US Food and Drug Administration [56]. According to the Brazilian Health Regulatory Agency (ANVISA) data, safrole use is allowed nationwide as a flavoring in products containing mace and nutmeg [57]. Regulation (EC) No. 1334/2008 of the European Parliament and of the Council [58] states that plant substances, such as safrole, can be used in foods when they are an intrinsic component of the flavoring, but using pure safrole is prohibited. Thus, flavorings with safrole in their composition can be added to alcoholic beverages, meat, poultry, fish, soups, and sauces.

The reduction in *C. maculatus* oviposition can be attributed to the essential oil components, which may have acted during the reproductive phase, hindering copulation or affecting physiological processes in mated females, inhibiting egg-laying. Oviposition decline may also be associated with the mortality of adult insects at the lethal concentrations, which led to less progeny emergence and, consequently, little egg production.

The essential oil residual effect may have affected the young stages of *C. maculatus* with ovicidal and larvicidal activities, which reduced the number of adults, as observed by Oliveira et al. [13]. These authors investigated the repellency of six essential oils to *C. maculatus* in free-choice bioassays at 500 µL/kg of cowpeas. They found that the essential oils of *P. hispidinervum* and *P. aduncum* diminished oviposition by 45 and 66% and emergence by 44 and 69%, respectively. In this way, they confirmed the insecticidal activity of the genus *Piper* on adult insect reproduction and early-stage development. Babarinde et al. [59] verified the insecticidal activity of the essential oil of *P. guineense*, applied by contact, on *C. maculatus* oviposition and emergence, in three cowpea varieties. They noticed that the variety and the essential oil of *P. guineense* had adverse effects on *C. maculatus* oviposition, progeny emergence (F1 and F2), and reproductive efficiency, in addition to reducing the percentage of grains damaged by the insects. Moreover, safrole concentration in the PHEO significantly decreased to less than 1% after five days of treatment by contact in stored cowpeas [19].

PHEO kept the quality of cowpea beans for 50 days of storage due to its toxicity to *C. maculatus* via different contamination routes. At all PHEO concentrations tested, grain quality was superior to that of the control treatment, and there was practically no weevil emergence.

The increase in grain water content is directly linked with less grain quality, thus being the parameter, most commonly used in quality assessments. Its increase is directly related to the biological activity of insects, mites, and fungi, in addition to inadequate storage [60,61,62]. In this study, the water content of the grains was preserved at all the PHEO concentrations evaluated.

The depletion of grain dry matter causes mass loss and bulk weight reduction. The feeding habits of the insect pests consist of perforating the tegument and consuming the embryo and cotyledons [29,63,64]. There was a minor mass loss, and bulk weight remained close to the initial value at the PHEO concentrations analyzed.

The diminution in grain germination capacity is an indirect effect of insect pests, as they feed on the embryo [61,64,65]. At all PHEO concentrations tested, germination was close to the initial value, whereas it was reduced in the control treatment.

PHEO has insecticidal potential against *C. maculatus* plaguing cowpea grains. Besides causing a lethal response to adults, it lowered the oviposition and progeny rates of the bruchid, even at Kovats sublethal concentrations, in the fumigation and contact bioassays. Overall, the present study results indicated that PHEO, rich in safrole (0.85 mg/L), may be employed for managing insect pests due to its toxicity. In addition, it can be included in management strategies aiming at minimizing the evolution of resistance to synthetic insecticides, which have been used continuously and indiscriminately for more than four decades. Rotating insecticides can substantially slow down resistance development [16,66]. It is also worth remarking that, under storage conditions, sublethal exposure to insecticides may occur. However, exposure of cowpea to sublethal PHEO concentrations did not compromise grain protection against *C. maculatus*.

It is important to mention that there is a restriction on the use of safrole in food. This restriction is because safrole and, especially, the products of its oxidation are carcinogenic [67,68]. In this scenario, safrole is classified among the substances generally prohibited from direct addition to or use as human food, according to the Food and Drug Administration [69]. Thus, future research is needed to determine, for example, the residual concentration of safrole during storage in treated grains.

## 5. Conclusions

PHEO, rich in safrole (0.85 mg/L), has insecticidal potential against *C. maculatus* plaguing cowpea beans. This research proved its fumigant and contact toxicity to adult individuals. The essential oil reduced the oviposition rate and insect progeny development while preserving grain quality after 50 days of storage, in both application forms.

The present study evidenced the insecticidal properties of PHEO, which can be added to the literature as a potential option to maintain the quality of stored cowpea beans by inducing insect pest mortality and inhibiting their development. Also, the low residual persistence in stored grains makes PHEO a safe alternative to treating food products.

## Figures and Tables

**Table 1:** Chemical composition and relative concentrations of the compounds in *P. hispidinervum* essential oil, as identified by gas chromatography coupled with mass spectrometry (GC-MS).

Compound	RI ^a^ (Literature)	RI ^b^ (Calculated)	Relative %
p-Cymen-8-ol	1179	1184	1.20 ± 0.02
Safrole	1285	1292	93.00 ± 0.72
(E)-Caryophyllene	1417	1415	0.69 ± 0.02
Bicyclogermacrene	1500	1493	2.05 ± 0.04
n-Pentadecane	1500	1498	1.60 ± 0.30
Spatulenol	1577	1573	1.46 ± 0.20

^a^ Relative retention index according to Adams [45] or the NIST-14 spectral library; and ^b^ retention index experimentally determined via a homologous series of alkanes C7–C30 (Kovats index) (Adams, 2007).

## Data Availability

The data presented in this study are available on request to the corresponding author.

## References

[1] Bakkali F., Averbeck S., Averbeck D., Idaomar M. (2008). Biological effects of essential oils—A review. Food Chem. Toxicol..

[2] Nenaah G.E., Ibrahim S.I., Al-Assiuty B.A. (2015). Chemical composition, insecticidal activity and persistence of three Asteraceae essential oils and their nanoemulsions against *Callosobruchus maculatus* (F.). J. Stored Prod. Res..

[3] Wang Y., Zhang L.T., Feng Y.X., Zhang D., Guo S.S., Pang X., Du S.S. (2019). Comparative evaluation of the chemical composition and bioactivities of essential oils from four spice plants (Lauraceae) against stored-product insects. Ind. Crops Prod..

[4] Idoko J.E., Ileke K.D. (2020). Comparative evaluation of insecticidal properties of essential oils of some selected botanicals as bio-pesticides against Cowpea bruchid, *Callosobruchus maculatus* (Fabricius) [Coleoptera: Chrysomelidae]. Bull. Natl. Res. Cent..

[5] Zimmermann R.C., de Carvalho Aragao C.E., de Araújo P.J.P., Benatto A., Chaaban A., Martins C.E.N., Zawadneak M.A. (2021). Insecticide activity and toxicity of essential oils against two stored-product insects. Crop Prot..

[6] Cerón D.A.C., de Alencar E.R., Faroni L.R.D.A., Silva M.V.D.A., Salvador D.V. (2023). Toxicity of allyl isothiocyanate applied in systems with or without recirculation for controlling *Sitophilus zeamais, Rhyzopertha dominica*, and *Tribolium castaneum* in corn grains. J. Sci. Food Agric..

[7] Bumbulytė G., Būdienė J., Būda V. (2023). Essential Oils and Their Components Control Behaviour of Yellow Mealworm (*Tenebrio molitor)* Larvae. Insects.

[8] Kavallieratos N.G., Eleftheriadou N., Boukouvala M.C., Skourti A., Filintas C.S., Gidari D.L.S., Maggi F., Rossi P., Drenaggi E., Morshedloo M.R. (2024). Exploring the efficacy of four apiaceae essential oils against nine stored-product pests in wheat protection. Plants.

[9] Araújo A.M.N., Faroni L.R.D.A., de Oliveira J.V., do Amaral Ferraz Navarro D.M., Breda M.O., França S.M. (2017). Lethal and sublethal responses of *Sitophilus zeamais* populations to essential oils. J. Pest Sci..

[10] Vilela A.D.O., Faroni L.R., Sousa A.H., Pimentel M.A., Gomes J.L. (2020). Toxicological and physiological effects of allyl isothiocyanate upon *Callosobruchus maculatus*. J. Stored Prod. Res..

[11] Dutra K.A., de Oliveira J.V., Navarro D.M.D.A.F., Barbosa D.R.S., Santos J.P.O. (2016). Control of *Callosobruchus maculatus* (F.) (Coleoptera: Chrysomelidae: Bruchinae) in *Vigna unguiculata* (L.) WALP. with essential oils from four *Citrus* spp. plants. J. Stored Prod. Res..

[12] Coitinho R.L.B.D.C., Oliveira J.V.D., Gondim Junior M.G.C., Câmara C.A.G.D. (2011). Toxicidade por fumigação, contato e ingestão de óleos essenciais para *Sitophilus zeamais* Motschulsky, 1885 (Coleoptera: Curculionidae). Ciênc. Agrotecnol..

[13] Oliveira J.V.D., França S.M.D., Barbosa D.R., Dutra K.D.A., Araujo A.M.N.D., Navarro D.M.D.A.F. (2017). Fumigation and repellency of essential oils against *Callosobruchus maculatus* (Coleoptera: Chrysomelidae: Bruchinae) in cowpea. Pesqui. Agropecuária Bras..

[14] Giunti G., Palermo D., Laudani F., Algeri G.M., Campolo O., Palmeri V. (2019). Repellence and acute toxicity of a nano-emulsion of sweet orange essential oil toward two major stored grain insect pests. Ind. Crops Prod..

[15] Santos J.C., Faroni L.R.A., Sousa A.H., Guedes R.N.C. (2011). 2011. Fumigant toxicity of allyl isothiocyanate to populations of the red flour beetle *Tribolium castaneum*. J. Stored Prod. Res..

[16] Souza L.P., Faroni L.R.D.A., Lopes L.M., de Sousa A.H., Prates L.H.F. (2018). Toxicity and sublethal effects of allyl isothiocyanate to *Sitophilus zeamais* on population development and walking behavior. J. Pest Sci..

[17] Pavela R., Benelli G. (2016). Essential oils as ecofriendly biopesticides? Challenges and constraints. Trends Plant Sci..

[18] Smith G.H., Roberts J.M., Pope T.W. (2018). Terpene based biopesticides as potential alternatives to synthetic insecticides for control of aphid pests on protected ornamentals. Crop Prot..

[19] Ferraz M.S.S., Faroni L.R.D.A., Heleno F.F., de Sousa A.H., Prates L.H.F., Rodrigues A.A.Z. (2021). Method validation and evaluation of safrole persistence in cowpea beans using headspace solid-phase microextraction and gas chromatography. Molecules.

[20] Negreiros J.R.D.S., Miqueloni D.P. (2015). Caracterização morfológica e fitoquímica de populações de *Piper hispidinervum* DC. e *Piper aduncum* L. no Acre. Ceres.

[21] Basak S., Guha P. (2017). Use of predictive model to describe sporicidal and cell viability efficacy of betel leaf (*Piper betle* L.) essential oil on *Aspergillus flavus* and *Penicillium expansum* and its antifungal activity in raw apple juice. LWT-Food Sci. Techonol..

[22] Branquinho L.S., Santos J.A., Cardoso C.A.L., da Silva Mota J., Junior U.L., Kassuya C.A.L., Arena A.C. (2017). Anti-inflammatory and toxicological evaluation of essential oil from *Piper glabratum* leaves. J. Ethnopharmacol..

[23] Gottlieb O.R., Koketsu M., Magalhães M.T., Maia J.G.S., Mendes P.H., Da Rocha A.I., Wilberg V.C. (1981). Óleos essenciais da Amazônia VII. Acta Amaz..

[24] Rossa G.E., Almeida R.N., Vargas R.M.F., Cassel E., Moyna G. (2018). Sequential extraction methods applied to *Piper hispidinervum*: An improvement in the processing of natural products. Can. J. Chem. Eng..

[25] Andrés M.F., Rossa G.E., Cassel E., Vargas R.M.F., Santana O., Díaz C.E., González-Coloma A. (2017). Biocidal effects of *Piper hispidinervum* (Piperaceae) essential oil and synergism among its main components. Food Chem. Toxicol..

[26] Ramos C.S., Barbosa Q.P. (2014). Metabolism of safrole by *Heraclides thoas brasiliensis* (Papilionidae). J. Lepid. Soc..

[27] Sohilait H.J., Kainama H. (2016). Synthesis of 1-(3, 4-methylenedioxyphenyl)-1-butene-3-one from safrole. Eur. J. Pure Appl. Chem..

[28] Ahn J.E., Zhou X., Dowd S.E., Chapkin R.S., Zhu-Salzman K. (2013). Insight into hypoxia tolerance in cowpea bruchid: Metabolic repression and heat shock protein regulation via hypoxia-inducible factor 1. PLoS ONE.

[29] Akami M., Chakira H., Andongma A.A., Khaeso K., Gbaye O.A., Nicolas N.Y., Nukenine E.N., Niu C.Y. (2017). Essential oil optimizes the susceptibility of *Callosobruchus maculatus* and enhances the nutritional qualities of stored cowpea *Vigna unguiculata*. R. Soc. Open Sci..

[30] Opit G.P., Thoms E., Phillips T.W., Payton M.E. (2016). Effectiveness of sulfuryl fluoride fumigation for the control of phosphine-resistant grain insects infesting stored wheat. J. Econ. Entomol..

[31] Arora S., Srivastava C. (2021). Locational dynamics of concentration and efficacy of phosphine against pulse beetle, *Callosobruchus maculatus* (Fab). Crop Prot..

[32] Pimentel M.A.G., Faroni L.R.D.A., Tótola M.R., Guedes R.N.C. (2007). Phosphine resistance, respiration rate and fitness consequences in stored-product insects. Pest Manag. Sci..

[33] Holloway J.C., Falk M.G., Emery R.N., Collins P.J., Nayak M.K. (2016). Resistance to phosphine in *Sitophilus oryzae* in Australia: A national analysis of trends and frequencies over time and geographical spread. J. Stored Prod. Res..

[34] Tay W.T., Beckett S.J., De Barro P.J. (2016). Phosphine resistance in Australian *Cryptolestes* species (Coleoptera: Laemophloeidae): Perspectives from mitochondrial DNA cytochrome oxidase I analysis. Pest Manag. Sci..

[35] Nayak M.K., Jagadeesan R., Singarayan V.T., Nath N.S., Pavic H., Dembowski B., Ebert P.R. (2021). First report of strong phosphine resistance in stored grain insects in a far northern tropical region of Australia, combining conventional and genetic diagnostics. J. Stored Prod. Res..

[36] Santos J.C., Faroni L.R.D.A., de Oliveira Simões R., Pimentel M.A.G., Sousa A.H. (2009). Toxicity of pyrethroids and organophosphorus insecticides to Brazilian populations of *Sitophilus zeamais* (Coleoptera: Curculionidae). Biosci. J..

[37] Gbaye O.A., Oyeniyi E.A., Ojo O.B. (2016). Resistance of *Callosobruchus maculatus* (F.) (Coleoptera: Bruchidae) populations in Nigeria to dichlorvos. Jordan J. Biol. Sci..

[38] Hagstrum D.W., Phillips T.W. (2017). Evolution of stored-product entomology: Protecting the world food supply. Annu. Rev. Entomol..

[39] Mir S.A., Mir M.B., Shah M.A., Hamdani A.M., Sunooj K.V., Phimolsiripol Y., Khaneghah A.M. (2023). New prospective approaches in controlling the insect infestation in stored grains. J. Asia-Pac. Entomol..

[40] Sutar S.A., Thirumdas R., Chaudhari B.B., Deshmukh R.R., Annapure U.S. (2021). Effect of cold plasma on insect infestation and keeping quality of stored wheat flour. J. Stored Prod. Res..

[41] Nasr G.M., Taha E.K.A., Hamza A.M., Negm E.A., Eryan N.L., Noureldeen A., Darwish H., Zayed M.Z., Elnabawy E.S.M. (2022). Gamma radiation: An eco-friendly control method for the rice weevil, *Sitophilus oryzae* (L.) (Coleoptera: Curculionidae). Biology.

[42] Ramírez-Rojas N.Z., Cerón-García A., Salas-Araiza M.D., Estrada-García H.J., Rojas-Laguna R., Sosa-Morales M.E. (2020). Radio frequency heating against *Sitophilus zeamais* Motschulsky in white maize. J. Stored Prod. Res..

[43] Brasil Ministério da Agricultura (2009). Pecuária e Abastecimento. Regras Para Análise de Sementes.

[44] Abbott W.S. (1925). A method for computing the effectiveness of an insecticide. J. Econ. Entomol..

[45] Adams R.P. (2007). Identification of Essential Oil Components by Gas Chromatography/Mass Spectrometry.

[46] Paes J.L., Faroni L.R., Martins M.A., Dhingra O.D., Silva T.A. (2011). Diffusion and sorption of allyl isothiocyanate in the process of fumigation of maize. Rev. Bras. Eng. Agrícola Ambient..

[47] Lu Y., Zhong J., Wang Z., Liu F., Wan Z. (2013). Fumigation toxicity of allicin against three stored product pests. J. Stored Prod. Res..

[48] Mossi A.J., Zanella C.A., Kubiak G., Lerin L.A., Cansian R.L., Frandoloso F.S., Treichel H. (2014). Essential oil of *Ocotea odorifera*: An alternative against *Sitophilus zeamais*. Renew. Agric. Food Syst..

[49] Huang Y., Ho S.H., Kini R.M. (1999). Bioactivities of safrole and isosafrole on *Sitophilus zeamais* (Coleoptera: Curculionidae) and *Tribolium castaneum* (Coleoptera: Tenebrionidae). J. Econ. Entomol..

[50] Sauter I.P., Rossa G.E., Lucas A.M., Cibulski S.P., Roehe P.M., da Silva L.A.A., von Poser G.L. (2012). Chemical composition and amoebicidal activity of *Piper hispidinervum* (Piperaceae) essential oil. Ind. Crops Prod..

[51] Clemes S.M., Santos T.G., Rebelo R.A., Laps R.R., Pescador R. (2015). Seasonality and hydrodistillation time effects on the yield and chemical composition of leaves essential oil of *Piper mikanianum* (Kunth) Steudel. Eclét. Quím. J..

[52] Rivera P.N., Mosquera T., Baldisserotto A., Abad J., Aillon C., Cabezas D., Manfredini S. (2015). Chemical composition and in-vitro biological activities of the essential oil from leaves of *Peperomia inaequalifolia* Ruiz & Pav. Am. J. Essent. Oils Nat. Prod..

[53] Simić A., Soković M.D., Ristić M., Grujić-Jovanović S., Vukojević J., Marin P.D. (2004). The chemical composition of some Lauraceae essential oils and their antifungal activities. Phytother. Res..

[54] Chan S.C., Choong Y.M., Weng S.H. (2018). Rapid Method for The Gas Chromatographic quantitative analysis to determinate safrole in commercial essential oils. J. Cosmet. Sci..

[55] Song X., Yin Z., Ye K., Wei Q., Jia R., Zhou L., Lv C. (2014). Anti-hepatoma effect of safrole from *Cinnamomum longepaniculatum* leaf essential oil in vitro. Int. J. Clin. Exp. Pathol..

[56] Shaaya E., Kostyukovysky M. (2006). Essential oils: Potency against stored product insects and mode of action. Stewart Postharvest Rev..

[57] ANVISA (2007). Agência Nacional de Vigilância Sanitária.

[58] European Union (2008). Regulation (EC) No. 1334, 16 de dezembro de 2008 of the European Parliament and of the Council. Off. J. Eur. Union.

[59] Babarinde S.A., Esan E.O., Olatunde O.Z., Ajayi D.S., Olaniyi J.P. (2017). Combination of *Piper guineense* essential oil with cowpea varietal resistance in control of cowpea seed beetle, *Callosobruchus maculatus* (F.) (Coleoptera: Chrysomelidae: Bruchinae). J. Northeast Agric. Univ..

[60] Mutungi C., Affognon H.D., Njoroge A.W., Manono J., Baributsa D., Murdock L.L. (2015). Triple-layer plastic bags protect dry common beans (*Phaseolus vulgaris*) against damage by *Acanthoscelides obtectus* (Coleoptera: Chrysomelidae) during storage. J. Econ. Entomol..

[61] Freitas R.S., Faroni L.R.A., Sousa A.H. (2016). Hermetic storage for control of common bean weevil, *Acanthoscelides obtectus* (Say). J. Stored Prod. Res..

[62] Silva M.G., Silva G.N., Sousa A.H., Freitas R.S., Silva M.S., Abreu A.O. (2018). Hermetic storage as an alternative for controlling *Callosobruchus maculatus* (Coleoptera: Chrysomelidae) and preserving the quality of cowpeas. J. Stored Prod. Res..

[63] Njoroge A.W., Affognon H.D., Mutungi C.M., Manono J., Lamuka P.O., Murdock L.L. (2014). Triple bag hermetic storage delivers a lethal punch to *Prostephanus truncatus* (Horn)(Coleoptera: Bostrichidae) in stored maize. J. Stored Prod. Res..

[64] Vales M.I., Rao G.R., Sudini H., Patil S.B., Murdock L.L. (2014). Effective and economic storage of pigeonpea seed in triple layer plastic bags. J. Stored Prod. Res..

[65] Hamdi S.H., Abidi S., Sfayhi D., Dhraief M.Z., Amri M., Boushih E., Jemâa J.M.B. (2017). Nutritional alterations and damages to stored chickpea in relation with the pest status of *Callosobruchus maculatus* (Chrysomelidae). J. Asia-Pac. Entomol..

[66] McKenzie C.L., Byford R.L. (1993). Continuous, alternating, and mixed insecticides affect development of resistance in the horn fly (Diptera: Muscidae). J. Econ. Entomol..

[67] Bogusz M.J., Al-tufail M., Bogusz M.J. (2008). Toxicological aspects of herbal remedies. Forensic Science: Handbook of Analytical Separations.

[68] Clarke S., Clarke S. (2008). Families of compounds that occur in essential oils. Essential Chemistry for Aromatherapy.

[69] FDA—Food Drug and Administration PART 189—Substances Prohibited from Use in Human Food. https://www.accessdata.fda.gov/scripts/cdrh/cfdocs/cfcfr/cfrsearch.cfm?fr=189.180.

